# Mass Cultivation of Microalgae: II. A Large Species Pulsing Blue Light Concept

**DOI:** 10.3390/biotech12020040

**Published:** 2023-05-17

**Authors:** Hans Chr. Eilertsen, Jo Strømholt, John-Steinar Bergum, Gunilla Kristina Eriksen, Richard Ingebrigtsen

**Affiliations:** 1 Norwegian College of Fishery Science, UiT The Arctic University of Norway, N-9037 Tromsø, Norway; gunilla.eriksen@uit.no (G.K.E.); richard.a.ingebrigtsen@uit.no (R.I.); 2 Finnfjord AS, N-9305 Finnsnes, Norway; jos@finnfjord.no (J.S.); johnb@finnfjord.no (J.-S.B.)

**Keywords:** marine microalgae, diatoms, photobioreactor, illumination, flashing blue light

## Abstract

If mass cultivation of photoautotrophic microalgae is to gain momentum and find its place in the new “green future”, exceptional optimizations to reduce production costs must be implemented. Issues related to illumination should therefore constitute the main focus, since it is the availability of photons in time and space that drives synthesis of biomass. Further, artificial illumination (e.g., LEDs) is needed to transport enough photons into dense algae cultures contained in large photobioreactors. In the present research project, we employed short-term O_2_ production and 7-day batch cultivation experiments to evaluate the potential to reduce illumination light energy by applying blue flashing light to cultures of large and small diatoms. Our results show that large diatom cells allow more light penetration for growth compared to smaller cells. PAR (400–700 nm) scans yielded twice as much biovolume-specific absorbance for small biovolume (avg. 7070 μm^3^) than for large biovolume (avg. 18,703 μm^3^) cells. The dry weight (DW) to biovolume ratio was 17% lower for large than small cells, resulting in a DW specific absorbance that was 1.75 times higher for small cells compared to large cells. Blue 100 Hz square flashing light yielded the same biovolume production as blue linear light in both the O_2_ production and batch experiments at the same maximum light intensities. We therefore suggest that, in the future, more focus should be placed on researching optical issues in photobioreactors, and that cell size and flashing blue light should be central in this.

## 1. Introduction

Due to their uniquely high growth rates and ability to produce nutritious biomass, photoautotrophic microalgae are considered by many to be the “green gold” of the future [1,2,3,4,5,6,7,8,9]. The potential products and services that can be derived from the algae biomass range from niche, high-value products (e.g., pigments, cosmeceuticals and pure omega-3 oil), to bulk materials (e.g., biofuel, nutraceuticals, functional food, livestock and fish feed) [10,11,12], to resource recovery from waste streams (e.g., precious metals and/or rare earths from waste streams) [13,14]. In the natural environment, microalgae produce more than half the O_2_ on earth in the process of converting CO_2_ to biomass by photosynthesis. This makes them powerful industrial fume CO_2_ sequestration agents [15,16]. Mass cultivation of microalgae has had industrial focus at least since the early 20th century [17,18,19,20]. Despite this, the global annual production is quite meagre and today probably only adds up to ca. 25,000–35,000 tons [21,22]. This is by no means sufficient to satisfy the need for protein and lipids in current and future aquaculture feed production. For example, Norwegian salmon production is projected to be ca. 5 million tons in 2050 [23]. To achieve this, at least 6 million tons of dry feed is needed (i.e., >200 times today’s global microalgae production). 

The underlying reason why microalgae mass cultivation has not gained sufficient momentum is that the production is expensive and complex. Key constraints in the production process relate to reactor design, contamination, temperature variations, gas exchange and especially illumination [24,25,26,27,28,29]. For obvious reasons, mass cultivation of microalgae takes place at concentrations much higher than during phytoplankton bloom events in the sea. Self-shading therefore becomes a problem, and optimal utilization of natural and artificial light become central issues in the cultivation process [25,26,27,28,30,31]. To achieve maximum conversion of available photons to biomass at high algae concentrations, the main factors that need to be considered are algae photosynthetic efficiency, photoperiod, light spectrum and intensity, and also absorption and scattering of light in the cultivation medium. 

One issue that has not had much focus is that the size of the microalgae can influence the biomass-specific absorption and scattering in such a manner that the same biomass concentration of large cells can have longer optical depths than small cells. As an organism gets bigger, the diameter, area and volume increase with first, second and third potency, respectively. The result of this is that large organisms have less self-shadowing per biomass unit than small organisms. In optical terms this is a result of the “package” effect [32,33]. It is obvious that this effect is modified by the fullness of material in the microalgae. Published and unpublished results though demonstrate that large microalgae cells can have longer optical depths per biomass unit than smaller ones [16,34,35,36,37]. Due to this, large cells potentially have more photons available for growth. This contrasts with the fact that most commercially cultivated microalgae are small green and blue-green species varying in size around 10 μm (e.g., *Chlorella*, *Spirulina*, *Daniella*, *Aphanizomenon*, *Haematococcus*, *Crypthecodinium*, *Schizochytrium*). In addition, *Schizochytrium* is not a photosynthesizing alga, but rather a single-celled heterotroph within the phylum Stramenopiles. Comparably, candidates from the diatom group range from 10 to >500 μm. This represents a range in cell surface area from 500 to 1 × 10^6^ μm^2^ and a volume range from 800 to 100 × 10^6^ μm^3^. In terms of potential absorption/diffusion of light, it is important to note that between diameters of 10 to 500 μm, the surface area increases 2000 times while the volume increases 125,000 times. It may therefore be well worth focusing on the potentially lowered self-shading abilities of large cells in microalgae mass cultivation initiatives. 

Apart from the importance of the cell diameter effect on optical penetration for obtaining efficient utilization of light, choosing the optimal light spectrum, intensity and photoperiod for the cultivated microalgae is also important. Photosynthesis is not “linear” but functions in pulses, and there are indications that synthesis of biomass and photosynthetic efficiency may be maintained (or sometimes even increased) with flashing light [38,39]. 

The present investigation is a continuation of a diatom mass cultivation initiative run by the ferrosilicon producing factory Finnfjord AS and UiT The Arctic University of Norway. A description of the project and some results are available in a previous publication [16]. Mass cultivation of photoautotrophs in large volumes is, as mentioned, challenged by illumination (self-shadowing) problems. The present investigation was therefore designed to reveal if energy (illumination) savings could be obtained by focusing on large cells, appropriate light spectrum and high-frequency flashing light. To investigate this, we applied both short-term (minutes) small-volume O_2_ production experiments and conventional large-volume batch (60 L) cultivation experiments.

## 2. Materials and Methods

In our experiments, we cultivated a diatom species (*Porosira glacialis*) utilized earlier in the large-scale mass cultivation initiative at Finnfjord AS [16]. The inocula used in all experiments were adapted to 8 °C and scalar light intensity 20 μmol m^−2^ s^−1^ for at least three days. PAR (Photosynthetic Active Radiation, 400–700 nm) light intensity was measured with a Biospherical (San Diego, CA, USA) QSL-100 instrument or a LI-COR (Cambridge, UK) LI-180 (cosine) spectrometer. Light intensity vs. wavelength was scanned with a LI-COR (UK) LI-180 (cosine, 380–780 nm) spectrometer. When converting measurements from μmol quanta m^−2^ s^−1^ to Wm^−2^ we multiplied with 0.2614 [40], and we used calculated biovolumes as a proxy for biomass. DW to biovolume ratio was checked for small vs. large cells.

### 2.1. Dry Weight vs. Cell Size vs. Biovolume-Specific Absorbance

For analysis of biovolume vs. DW (dry weight), large and small cells of the same species from the stock culture collection at Finnfjord AS (diameter 17.045 μm and 33.64 μm, height 30.75 μm and 20.99 μm and concentration ca. 10^6^ cells L^−1^) were scanned for absorbance in 5 cm quartz cuvettes in a spectrophotometer (VWR UV/Visible spectrophotometer, UV-6300PC). We measured 33 cells of each size group at 400× magnification in a Zeiss inverted microscope, and each culture was scanned 6 times. Differences in absorption between scans were negligible, so we applied means of measurements in the further interpretations. Following this, 3 × 200 mL of each cell size culture was collected onto 47 mm burnt GF/C filters. To remove salt from culture seawater that could interfere with weight, each filter was rinsed with 10 mL Millipore-treated freshwater prior to drying. Prior to the experiment, each filter was weighted using a Sartorius Entris weight. After drying the filters for 2 h at 60 °C (or until the weight was stable) in a Termaks KB temperature-controlled cabinet (Nordic Labtech AB, Fjärås, Sweden), they were re-weighted.

### 2.2. Diffuse Light Extinction vs. Cell Size

**Figure 1:**
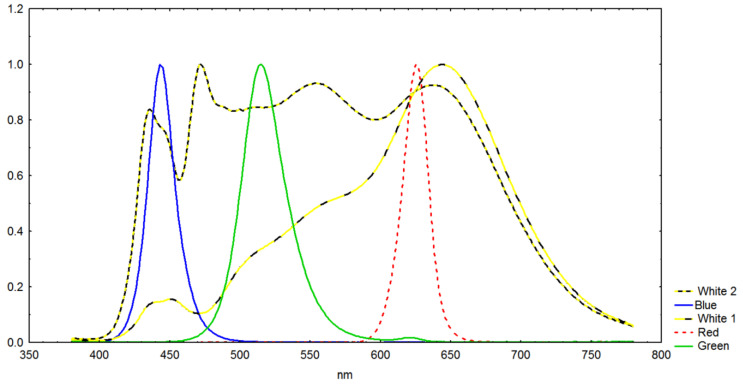
Light intensity (y-axis) vs. wavelength for the different illumination colors used during the linear and pulsed O_2_ production experiments. All measurements were at ca. 75% of maximum intensity, whereafter intensity values were normalized in that all values for each color were divided by the maximum intensity at that color.

Here, cell concentration, cell sizes and irradiance were measured during routine cultivation periods in a 300 m^3^ white coated glass fiber vertical column photobioreactor [16]. Cell concentrations were monitored daily with Leica or Zeiss inverted microscopes (200 or 400× magnification), while applying the Utermöhl [41] method on cells fixed with Lugol’s iodine solution [42] and using Nunc 4 well 1.9 mL cultivation chambers (settling time > 2 h). Cell size (diameter, height) was measured on at least 10 cells ca. once weekly. Light in the reactor was a mixture of natural and artificial (White 2, Figure 1) illumination. Light intensity was measured with a spherical quantum LI-COR LI-193 (UK) sensor at −0, 0.1, 0.2, 0.3, 0.4, 0.5 and 0.6 m depth. For ca. 20 samplings from the 300 m^3^ reactor, representing periods with different cell sizes, the diffuse extinction coefficients (*k*) between 0.2 and 0.6 m were calculated using the formula:

(1) $$k=\frac{lnI_{0}-lnI_{D}}{D}$$

where *I*_0_ and *I_D_* are light intensity at depths and *D* is distance in m between depths. Biomass proxy for each set of cell concentrations was biovolume as calculated from [43].

### 2.3. O_2_ Production vs. Spectrum vs. Linear or Pulsed Light

These experiments were performed using an in-house constructed, digitally controlled illumination rig with combinations of LEDs with different colors. The “face” of the rig where LEDS were mounted was 60 × 35 cm. The rig has an integrated power supply, and we can illuminate cultures (here in 500 mL glass beakers) with variable colors, intensities and either linear or pulse width modulated (PWM, 1 Hz to 1 kHz) light. The LEDs (with cooling fans) were controlled with a programmed Raspberry PI unit. The wavelength areas (spectra) applied by us in the experiments were Blue, Red, Green and two types of white (White 1, White 2). White 2 had more shortwave radiation than White 1 light (Figure 1) and the spectra were measured inside the beaker where cultures were illuminated.

When we compared linear and PWM light with the “same intensity”, this refers to the same intensity with LEDs on in PWM mode (irrespectively of frequency) as in linear (100% duty cycle) mode. In other words, the maximum intensity was always the same in linear and PWM mode.

The stock cultures we used were maintained in pasteurized f/10 growth medium [44] with additional silicate added (12.3 μmol Si(OH)_4_ L^−1^). Cells were relatively large ones (30–32 μm diameter). The cultures were adapted >3 days to 8 °C at 20 μmol m^−2^ s^−1^ LED with white light (White 2, Figure 1) measured incident onto the surface of 2 L Nunc plastic culture flasks, and the cultures were kept in a temperature regulated culture cabinet (Termaks KB series, Nordic Labtech AB). To obtain P vs. I (Photosynthesis vs. Irradiance) data, the microalgae cultures were exposed to light gradients in two ways:

Sequential exposure: Prior to experiments, the cultures were kept in dark for 1 h. The culture (400 mL) was then exposed for 3 min to different light intensities (10, 20, 35, 60, 110 μmol m^−2^ s^−1^), this being without pauses between exposures. For daylight (White 2 in Figure 1) this is equivalent to ca. 2.6, 5.2, 9.1, 15.6, 28.6 W m^−2^. Oxygen concentration was registered at the start and stop of the 3 min sessions, and the difference (increase) was recalculated to uptake minute^−1^ 10 mill cells L^−1^. This sequential method was meant to mimic the variable light intensities culture cells experience when mixed around in reactors. It also represents P vs. I responses which result from the prevailing light adaptation level of the cells, which are suitable for comparing different light spectra and intensities. The illumination gradient was determined from previously performed P vs. I ^14^C exposure experiments (own unpublished) that indicated an *I_opt_* light intensity below 95 μmol m^−2^ s^−1^ at the given temperature (8 °C) and light adaptation. Each of the P vs. I measurement series (to increasing intensity in the 10, 20, 35, 60, 110 μmol m^−2^ s^−1^ gradient) were repeated at least 3 times. All these exposures were performed using linear and 1, 10, 50, 100 and 500 Hz PWM light at all spectra in Figure 1.

Repeated exposure to the same light intensity: For each experiment series, the cultures were kept in the dark for one hour prior to treatment with 10 μmol m^−2^ s^−1^ intensity for one hour. Thereafter we exposed cultures and measured O_2_ production for three minutes and at least three times to the 10 μmol m^−2^ s^−1^ intensity. This procedure was repeated for the other (20, 35, 60, 110 μmol m^−2^ s^−1^) light intensities. These exposures were both to linear and 1, 10, 50, 100 and 500 Hz PWM light.

The illumination rig was placed 30 cm in front of the beaker that was ca. 30% submerged in water that circulated through a temperature regulated cooling device. Exposures were performed at 8 +/− 0.3 °C. Temperature, pH and O_2_ during each exposure were measured with a WTW Multi 360 m instrument, a WTWSenTix 940 IDS probe and a CellOx 325 sensor (Xylem Analytics, Weilheim, Germany). Prior to the experiments, pH and O_2_ saturation were adjusted to ca. 7.0 and 80% by carefully bubbling N_2_ and CO_2_ into the culture beaker. During exposures pH and O_2_ values were kept below 7.5 and 90% and CO_2_ above 2.65 mgL^−1^.

### 2.4. Lipid Class Analysis

Extraction of lipid followed Jensen et al. [45] adapted from the Folch method [46], using dichloromethane:methanol (2:1 *v*/*v*) [47]. The biomass was extracted twice to maximize yield. The lipid class composition was analyzed by normal phase HPLC, using a Water e2795 separations module coupled to a Supelcosil TM LC-SI 5 mm (25 cm → 4.6 mm) column (Supelco HPLC products, Bellefonte, PA, USA) and set to a working temperature of 40 °C. The HPLC method used was modified from Abreu et al. [48]. The measurements did not allow for quantification of total lipid weight but only relative lipid class composition.

## 3. Results

### 3.1. Dry Weight vs. Cell Size vs. Biomass-Specific Absorbance

Biovolume for the large cells was ca. 12.5 times larger for small cells, while as shown in Table 1 biomass-specific DW, it was ca. 17% lower for the large than for the small cells.

**Figure 2:**
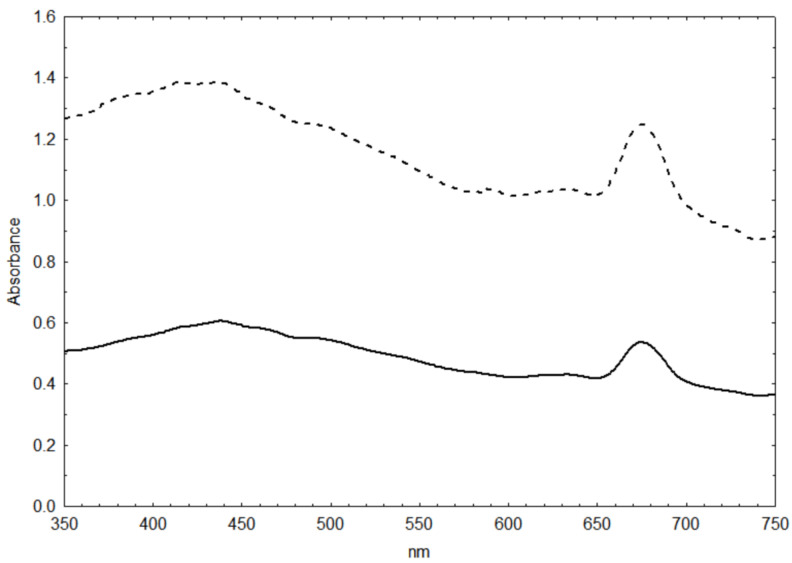
Biovolume-specific absorption of two diatom size groups; upper curve: small cells and lower curve: large cells.

Scans vs. wavelength revealed that small cells had > twice as high biovolume-specific absorbance than large cells (Figure 2).

### 3.2. Diffuse Light Extinction vs. Cell Size

The data applied here were from reactor culture samples with minimum and maximum cell diameters of 21 and 45 μm and means from 26.9 to 37.2 μm. To represent large and small cells in the computation of the diffuse extinction coefficient (*k*), we used light intensity data relating to cells with 24 and 42 μm frustule width. Mean height was 26.1 and 27.25 μm respectively.

**Figure 3:**
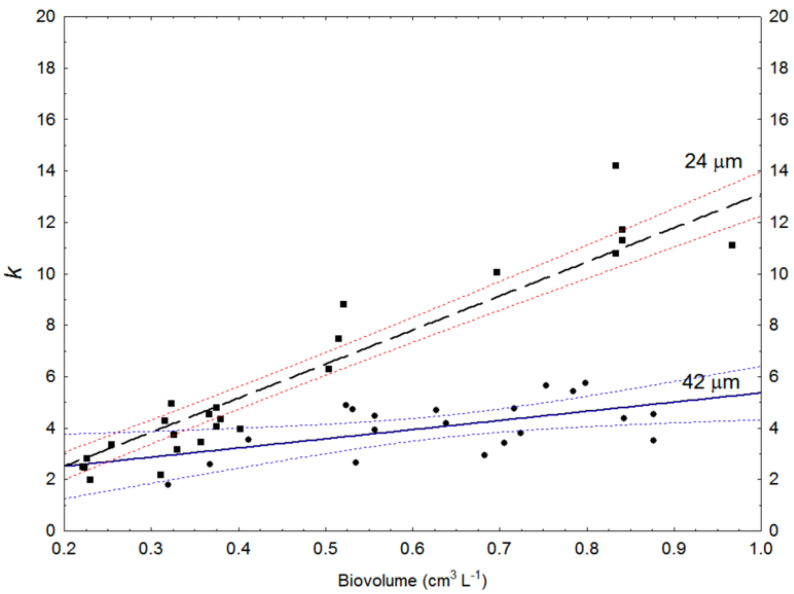
Diffuse light extinction coefficient (*k*) values for 42 and 24 μm diameter cells, calculated from measured scalar light at 0.2 and 0.6 m under the surface in a 300 m^3^ photobioreactor. Dotted lines are 95% confidence regression bands.

The resulting computed values demonstrates that *k* increased faster with biomass concentration for small (24 μm) cells than for large (42 μm) cells (Figure 3). For cell biomass concentration, 0.4 cm^3^ L^−1^ mean *k* was 5.8 for 24 μm cells and 3.3 for 42 μm ones, while at 0.8 cm^3^ L^−1^ biomass, *k* values were 10 and 4.5, respectively (Figure 3).

When large (42 μm) cells were applied, calculated light intensities at 0.5 m with biomass concentration 0.4 gL^−1^ were nearly four times higher compared to smaller (24 μm) ones. With biomass 0.8 cm^3^ L^−1^ and small cells, computed light was nearly absent at 1.0 m (Table 2).

### 3.3. O_2_ Production vs. Wavelength vs. Linear or Pulsed Light

To assure that measurements were mainly from the light-limited linear part (i.e., *I_opt_*, Photosynthesis maximum) of the P vs. I curve [49,50], we applied 110 μmol quanta m^−2^ s^−1^ light intensities and below.

**Figure 4:**
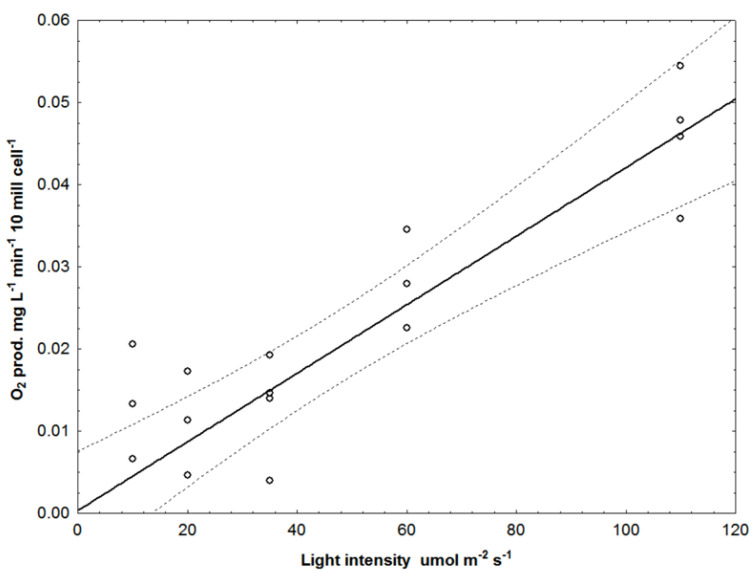
Oxygen production vs. blue linear light intensities (10, 20, 35, 60, 110 μmol quanta m^−2^ s^−1^). Dotted lines are 95% confidence regression bands. Data from sequential exposure.

Obvious outliers in our data sets were discarded by using a modified winsorization regression technique [51] with 80% cutoff. Here, data above the 90th percentile and below the 10th percentile were given the values of the upper and lower percentiles, exemplified in Figure 4 for blue light.

As described in the Material and Methods section, we tested linear vs. PWM illumination at the selected intensities (10, 20, 35, 60, 110 μmol quanta m^−2^ s^−1^) for five irradiance spectra (Blue, Green, White 1, White 2, Red, see Table 3). When reporting data in Table 3 we did not apply standard deviation (S.D.), since the measured O_2_ production values were responses to the application of varying light intensities.

The results from this test were (Table 3):

1. Mean O_2_ production for blue light was the highest and almost the same for linear and 100 Hz light.
2. White 1 spectrum had high O_2_ production and alpha values with PWM light.
3. Blue 100 Hz and linear light both had high O_2_ production and alpha values.
4. Repeated exposure to the same linear and 100 Hz blue light intensities (Table 4) yielded higher O_2_ production values than the 3 min light gradient exposures.

Dark respiration was also measured 3 times during these experiments (not shown) and varied between 2 and 4.5% of the mean O_2_ production. 

We did not apply reporting of standard deviation (S.D.) on the sequential data sets but only means. This is since the measured O_2_ production values varied with the light intensities applied. The results showed that the mean O_2_ production for blue light was the highest and almost the same for linear and 100 Hz light (Table 3).

White 1 also had both high O_2_ production and alpha values with PWM light. Further, it was blue 100 Hz light, apart from linear light, that had both high O_2_ production means and alphas. Dark respiration was also measured 3 times during these experiments (not shown) and varied between 2 and 4.5% of the mean O_2_ production.

Repeated exposure to the same linear and 100 Hz blue light intensities (Table 4) yielded higher O_2_ production values than the 3 min light gradient exposures.

Similar to the sequential exposures, blue linear and 100 Hz light yielded the same O_2_ production values. Here also, both linear and PWM light showed a clear P vs. I response where the largest increase occurred for irradiances between 20 and 35 μmol quanta m^−2^ s^−1^. The highest O_2_ values were at 210 μmol quanta m^−2^ s^−1^.

### 3.4. Microalgae Growth with Blue Linear and Pulsed Light

All growth vs. linear and 100 Hz PWM light experiments had clear exponential growth from the start (day 1) until they were harvested on day 7, except for the linear culture 2 (L2, Table 5), that for unknown reasons had stagnated growth on the last 2 days. Growth (doublings day^−1^) were therefore only from the first 5 days. The growth rates varied around 1 doubling day^−1^. Linear experiment 1 (L1) had the highest mean growth rate (1.23 doublings day^−1^), closely followed by PWM light 1 (P1) that had 1.09 doublings day^−1^. From the growth rate S.D. values, it appears that all growth rates are statistically indistinguishable (Table 5). This was likely caused by (normal) uneven growth between days. Correlation tests between growth and temperature, as well as oxygen and pH, revealed no meaningful correlations. We therefore conclude that linear and PWM blue light had the same effect on the growth rates measured from increase in cell concentrations.

### 3.5. Lipid Class Distribution

The only clear trend with respect to lipid class distribution was that PWM light triggered considerably lower TAG (triacylglycerol) production (mean 18.73) compared to linear light, and that this was compensated by increased amounts of MGDG (Table 6).

## 4. Discussion

Photoautotrophic microalgae surely have the potential to be the future’s “green gold” due to their high growth rates and nutritional value, combined with their high CO_2_ sequestering and O_2_ production abilities. In addition, they can be cultivated in area-saving reactors which would not compete for arable land. To reach a situation where microalgae biomass production can rival current agricultural crops (e.g., world soy production at 354,000,000 tons in 2020), it will be paramount to perform considerable optimizations of production processes. Due to self-shadowing, large cultivation volumes of dense cultures will need artificial illumination, and this comes at a cost [30]. Optimizations towards efficient and cost-effective illumination of the cultures combined with high photosynthetic efficiency is therefore of prime importance, and here no stone should be left unturned. The aspects we focus on here (large cells and blue flashing light) are not, to our knowledge, tested together and implemented in current microalgae biorefineries. The obvious reason why blue light is interesting is that it penetrates more easily and deeper into water/other media than longer wavelength light and it peaks around the maximum absorbance of Chla.

The k (diffuse extinction coefficient) values we calculated after having measured irradiance at different depths in the 300 m^3^ reactor clearly demonstrate that microalgae size can influence optical depths in dense cultures. The light intensities calculated in Table 2 are based on biovolume as a biomass proxy. It can here be argued that less biomass can be present per biovolume unit in large compared to smaller cells. Our routine samplings during cultivation sessions in the 300 m^3^ reactor have not revealed such traits (harvested biomass vs. biovolume), while our DW vs. biovolume ratio measurements (Table 1) revealed ca. 17% lower biovolume-specific DW for large cells. However, biovolume-specific scans revealed a doubling of absorbance for small cells compared to large ones, and in total this demonstrates 1.75 times higher DW-specific absorbance. Our routine samplings of cells of different sizes vs. measurements of irradiance should reflect reasonable “normal” cultivation sessions. Typical biovolume concentrations in such situations are 0.4–0.8 cm^3^ L^−1^, depending on cell size. If cells then have approximately the same biomass to biovolume ratio irrespective of size, at 1 m depth, 100 μmol m^−2^ s^−1^ will be reduced to 4.59 and 0.66 μmol m^−2^ s^−1^ for 42 and 24 μm cells, respectively, if biovolume is 0.8 cm^3^ L^−1^. Furthermore, at biovolume 0.4 cm^3^ L^−1^, if a cell is in an environment with artificial illumination units 2 m apart with constant mixing speed, the 42 μm cell would receive a mean irradiance of 26 μmol m^−2^ s^−1^ compared to 14 μmol m^−2^ s^−1^ with a 24 μm cell (applying Formula 1). Such self-shadowing aspects have only sporadically been described in biology-focused literature [31,32,33,34,35,36,37,38] and does not appear to be a theme in microalgae biorefinery texts [16]. Our results are based on experiments with the same diatom species, but we expect that the concept is potentially valid across broader taxonomies and should be tested for single cell photoautotrophs in general. Additionally, it should be noted that we have focused on light available to microalgae, and not how they photosynthesize/absorb and reflect photons. A well-known “dogma” related to microalgae size and metabolism is that the pace of metabolism decreases allometrically with increasing cell size. This is, however, not generally valid, and new experimental evidence indicates that biomass-specific production and growth rates can be comparable in both small and large cells [52]. Further, what is left out of these discussions is the question of whether the cells operate at maximum growth rates or not. In fact, this seldom happens in reactors. Another issue that can lead to misunderstandings is that in a commercial photobioreactor, it is usually light, rather than temperature or inorganic nutrients, that limits growth. This makes the optical conditions (here optical depth) in the reactor of prime importance. It is also important to note that in persistent cultivation of a species, the photosynthetic characteristics can co-evolve with cell volume [53]. We therefore conclude that, when cell specific biomass (DW) increases with size, increased cell size can increase the optical depth in the culture.

Measurement of O_2_ evolution has been widely used to quantify phytoplankton net production, both in natural samples and in laboratory experiments. Quantification of released O_2_ can be done using different methods to analyze the content in water (cultivation medium), both at the beginning and end of a defined illumination exposure period [54]. In laboratory experiments it is common to expose the microalgae culture to a gradient in light intensity and thereafter fit the data to a P vs. I model. Details in the experimental methods used vary considerably between studies [49,55]. In our experiments we applied a fast response electrochemical O_2_ sensor [56]. As we practiced it, the method measured net O_2_ production, while respiration was measured separately in dark conditions. As with most other methods used to measure photosynthesis, it has been debated if the method is useful to measure production, most often by comparing it to results from the ^14^C tracer method. How oxygen is measured also matters; Ryther and Vaccaro used a light/dark bottle method and Williams et al. used an electrode [57,58], and both found reasonably good agreements. Trampe et al. [59] observed variable correlations and attributed this to the fact that each method measures the activity of different reactions in the photosynthetic pathway. However, it is our conclusion that O_2_ production is a reliable biomass production proxy, both due to its tight coupling to photon harvesting [59] as well as the constant ratio to CO_2_ uptake [58]. Additionally, the electrode method is simple to use and it reacts ultra-fast to changes in O_2_ in the culture [60].

Our results showed that for sequential exposures, mean O_2_ production and alpha for blue light was the highest and almost the same for linear and 100 Hz light (Table 3). White 1 also had both high O_2_ production and alpha values. Dark respiration was measured 3 times during these experiments (not shown) and varied between 2 and 4.5% of the mean O_2_ production.

There are numerous observations concluding that microalgae and especially diatoms [61,62,63,64] have the most energy-efficient biomass synthesis in blue or blue-green (shortwave) light, i.e., at wavelengths where Chl*a* and other pigments have absorption maxima [65]. Also relevant in terms of diatom mass (biorefinery) cultivation is that blue light is essential for high light acclimation in diatoms [64]. This can play an important role in reactors where light is unevenly distributed and cells are mixed towards or away from high light intensities (from natural illumination or LEDs). The main reason for performing sequential 3 min exposures to increasing light intensities was to mimic the variable light intensities that algae cells experience when mixed in a vertical column photobioreactor. In a real cultivation situation, the cells will though experience much faster variations, i.e., mixing rates >0.1 m s^−1^ [16]. Our sequential exposure results must, in our opinion, be considered indicative of relative photosynthesis (growth) at linear and PWM light vs. different spectra (light colors) at the prevailing adaptation conditions. When we performed repeated exposures to the same linear and 100 Hz blue light intensities, we logged substantially higher O_2_ production values than the 3 min sequential light exposures. Similar to the sequential gradient exposures, blue linear and 100 Hz light had highly comparable O_2_ production values. Both linear and PWM light also had a P vs. I response. The fastest increase for blue light was up to irradiances between 20 and 35 μmol quanta m^−2^ s^−1^, but the highest O_2_ values were at 210 μmol quanta m^−2^ s^−1^. Even if the sequential experiments yielded lower mean O_2_ production (Table 3), the steepness (alpha) of the P vs. I curves were sometimes higher, and maximum O_2_ productions were more similar to repeated exposures to the same light. We believe this simply reflects the adaptation to 20 μmol m^−2^ s^−1^ that took place prior to the experiments. Repeated measurements with the same light also allowed cells to adapt to the selected intensity. In a large photobioreactor with submerged LEDs, the light intensities that the cells are exposed to will also be heavily influenced by reactor and illumination construction specific details [66], in total probably often varying between zero and 1000 μmol m^−2^ s^−1^ [16,67].

Photoautotrophic microalgae must both utilize available photons as efficiently as possible and avoid possible damaging effects of high light intensities. Induction of photoprotection may be rapid (<0.5 h) while low light adaptation is more of a slow process. Nymark et al. [68] observed a steady increase in the light saturation index from ca. 105 to 130 μmol m^−2^ s^−1^ after adaptation to 35 μmol m^−2^ s^−1^. However, during long-term cultivation experiments at low temperatures, Gilstad and Sakshaug [69] observed that maximum growth rates for our species took place in as little as 33 μmol m^−2^ s^−1^ light, partly in accordance with our O_2_ production results. This also confirms the common conception that in diatoms, and especially in arctic species, the photoadaptive strategy is tuned to low light environments [70,71].

Data that describe diatom photosynthetic response to fast-fluctuating light (as experienced by mixing in reactors) is scarce. A large part of these studies conclude that diatoms adapt fast and well to prevailing light regimes [62,72,73]. Our results, that sequential exposure to increasing light yields less O_2_ production than repeated exposure to the same light, unsurprisingly, may indicate that a homogenous light climate is to be preferred. Alternatively, this may reflect that the exposure conditions we applied cannot be directly transferred to large-scale reactor mixing conditions. We consider that the results in Table 3 were suited to discriminate O_2_ production between different spectra and linear and PWM light. We interpret the data in Table 4 as production ranges which are more relevant to the chosen light intensities in a reactor with homogenous illumination. The main conclusion here is that blue PWM 100Hz light has production yields comparable to linear blue (or other spectra) light at the same maximum light intensity.

A timely remark here is that it is of utmost importance to define clearly how light is measured to allow light climate vs. production to be precisely interpreted [74]. This includes descriptions of light meters used, discrimination between scalar and cosine measuring method, and if instruments were calibrated. An issue that can introduce misinterpretations and difficulties in comparing results is that when performing batch cultivation, cell concentrations will increase and make precise illumination difficult to describe.

Our 7 days’ growth experiments clearly confirm the results from the O_2_ production experiments: that growth rates are highly comparable for blue linear and 100 Hz PWM light. The mean obtained growth rates varied by around 1.0 doublings day^−1^ (Table 5) and the results were statistically indistinguishable. The main reason why this is interesting in a biorefinery context is that when applying 100 Hz square light, the quantity of photons delivered into the culture is only 50% of photons in linear mode. With dedicated power supplies this potentially can imply large energy savings. Maximum obtainable growth rate at the prevailing temperature (ca. 10 °C) is ca. 1.6 doublings day^−1^ according to Eppley [75]. If the maximum in Eppley shall be obtained, illumination, temperature, nutrients and CO_2_ supply must be optimal. Species-specific temperature optimums also play a role. During our experiments we believe that it was CO_2_ limitation (high pH) and possibly high O_2_ concentrations that limited growth somewhat, but our obtained growth rates are in the high end of what is usually obtainable at ca. 10 °C in culture. Our own cultivation experiments with *P. glacialis* have shown a maximum of around 8–10 °C, but it also decreases well down to 0 °C [76].

The use of high-frequency flashing LED illumination can be a novel and innovative method to mitigate light attenuation in photobioreactors [39]. Microalgae can have comparable or even higher photosynthetic rates when exposed to flashing compared to linear light [77,78]. One theory is that light pulses of 100 μs or shorter are stored in reaction centers and available to electron transport in the dark period, indicative of photosynthesis working close to its limits [37]. Our best production with PMW illumination was with much longer square pulses (5 ms at 100 Hz). Diatoms may also act differently from green and blue-green microalgae, for example, and some other investigations with microalgae have reported the same growth with 100 Hz (or lower) frequencies compared to linear light [79,80,81].

It is probable that short light duty cycles from ms and below only have minor intracellular effects while ms and longer cycles can have effects on e.g., lipid and amino acid composition [39,82,83,84]. This potentially can enable designed production of desired products.

Lipid class analysis of biomass from our blue linear vs. 100 Hz long-term growth experiments (Table 6) showed a significant (ca. 50%) decrease in the TAG (triacylglycerol) content in the 100 Hz samples, compared to the linear ones. This was compensated by increased amounts of MGDG (monogalactosyldiacylglycerol). Apart from that a decreased content of TAG in food and fish feed can be beneficial for e.g., cardiovascular health [85,86], this demonstrates that logging the chemical composition of the algae when manipulating PWM illumination is important.

The main result from our experiments is that high-frequency flashing light has the potential to significantly reduce energy use in LED-illuminated photobioreactors. If square flashing light is applied with dedicated cost-effective power supplies, light is only “turned on” during the duty cycles [87]. We suggest combining this with large cells and blue light. Other measures that can contribute to save energy used in reactors include cultivation of species with high photosynthetic efficiency or high Rubisco (CO_2_ uptake) specificity and genetic manipulation [88,89].

## 5. Conclusions

Our results showed that large diatom cells can make more photons available for growth compared to smaller cells, since biovolume-specific light absorbance was here (1.75 times) lower for large than for small cells. It is well known that blue light penetrates water more easily than light at longer wavelengths, but the effects on growth and chemical content is variable. In our production experiments we observed higher O_2_ production values for blue light compared to two types of white (natural), green and red light. Further, we observed similarly high values for blue 100 Hz pulsing light than for linear blue light. Ordinary batch cultivation experiments confirmed these results, i.e., we observed the same (ca. 1.0) doubling rates for pulsing and linear blue light. However, we observed a decrease (ca. 50%) in the lipid class TAG (triacylglycerol) when 100 Hz was applied. In sum, this highlights that potentially significant energy savings can be obtained by having a large focus on light quality (i.e., spectrum, pulsing) vs. cell size.

## Figures and Tables

**Table 1:** Cell size measurements, cell biovolume and biovolume-specific dry weight (DW).

Cell Diameter/S.D. μm	Cell Height/S.D. μm	Cell Concentration (Cells L^−1^)	Single Cell Biovolume (μm^3^)	Culture Biovolume-Specific DW (g cm^−3^)
17.045/1.15	30.758/1.27	8,922,760	7070	0.179
33.636/7.42	20.985/3.23	11,457,635	18,703	0.151

**Table 2:** Light intensity at 0.5 and 1.0 m depth computed from *k* values in Figure 3 using Equation (1).

Mean Cell Width (μm)	Biovolume (cm^3^ L^−1^)	Light Intensity μmol m^−2^ s^−1^ at Depth		
		0 m	0.5 m	1.0 m
24	0.4	100	8.16	0.66
42	0.4	100	21.23	4.59
24	0.8	100	0.67	0.005
42	0.8	100	10.53	1.11

**Table 3:** Mean O_2_ production (mg L^−1^ min^−1^ 10 mill cell^−1^) of sequential exposures to 10, 20, 35, 60, 110 μmol quanta m^−2^ s^−1^ (cosine) of different light spectra generated with LEDs working in linear and pulse width modulated (PWM) mode. Numbers in parentheses are alpha (mg O_2_ L^−1^ min^−1^ 10 mill cell^−1^ μmol quanta m^−2^ s^−1^) values, i.e., (linear) slope of initial light limited part of P vs. I curve. Cell concentrations were between 23 and 50 mill cells L^−1^, i.e., each exposure was at least performed sequentially (vs. increasing intensity) three times. The two highest mean O_2_ production values are marked green, and the two highest alpha values are yellow. Total *n* = 92.

Spectra	Unit–O_2_ Production	Linear	1 Hz	10 Hz	50 Hz	100 Hz	500 Hz
Blue	mg O_2_ L^−1^ min^−1^ 10 mill cell^−1^	0.022	0.016	0.012	0.012	0.020	0.016
Green		0.015	0.008	0.008	0.017	0.012	0.008
White 1		0.014	0.024	0.015	0.019	0.017	0.010
White 2		0.018	0.014	0.008	0.011	0.016	0.015
Red		0.012	0.009	0.015	0.006	0.013	0.005
	Unit–alpha values						
Blue	mg O_2_ L^−1^ min^−1^ 10 mill cell^−1^ μmol quanta m^−2^ s^−1^	0.00074	0.00056	0.00052	0.00027	0.00082	0.00055
Green		0.00064	0.00032	0.00028	0.00083	0.00039	0.00040
White 1		0.00052	0.00096	0.00054	0.00073	0.00077	0.00037
White 2		0.00059	0.00062	0.00037	0.00040	0.00067	0.00060
Red		0.00036	0.00038	0.00048	0.00035	0.00036	0.00015

**Table 4:** Mean O_2_ production (mg L^−1^ min^−1^ 10 mill cell^−1^) from repeated exposures to 10, 20, 35, 60, 110 μmol quanta m^−2^ s^−1^ (cosine) of blue light generated with LEDs working in linear and 100 Hz Pulse Width Modulated (PWM) mode. Cell concentrations were between 23 and 50 mill cells L^−1^.

Irradiance (μmol m^−2^ s^−1^) Blue Linear	*n*	Production (mg O_2_ L^−1^ min^−1^ 10 mill Cell^−1^)	S.D.
10	21	0.0337	0.0073
20	21	0.0433	0.0095
35	20	0.0467	0.0011
60	17	0.0508	0.0110
210	12	0.0591	0.0120
Mean		0.0467	
Blue PWM			
10	15	0.0374	0.0014
20	18	0.0485	0.0010
35	26	0.0461	0.0101
60	21	0.0466	0.0110
210	14	0.0554	0.0150
Mean		0.0468	

**Table 5:** Mean growth rate (doublings day^−1^, temperature, oxygen saturation) for 7-day cultivation periods. pH is from the last sampling day and measurements were performed on day 1, 2, 3, 4, 5 and 7. Standard deviations (95% ci) are in parentheses; L: linear; PWM: pulse width modulated blue irradiance.

Experiment	Doublings Day^−1^	Temperature (°C)	Oxygen (% Saturation)	pH
L1	1.23 (0.75)	9.64 (1.67)	99.2 (1.13)	8.42
L2	1.08 (0.70)	9.65 (1.09)	101.8 (2.20)	8.50
L3	0.72 (0.50)	8.86 (1.38)	99.6 (1.77)	8.48
Mean Linear	1.01			
P1	1.09 (0.34)	10.56 (0.86)	111.6 (1.16)	8.21
P2	0.83 (0.33)	10.04 (1.03)	100.78 (0.48)	8.46
P3	1.00 (0.35)	8.77 (1.33)	100.33 (4.59)	8.45
Mean PWM	0.97			

**Table 6:** Lipid class distribution from cultivation experiments with blue linear and PWM light (see Table 3). Amounts are relative where total lipid: 100; TAG: triacylglycerol; DAG: diacylglycerol; FFA: free fatty acid; MAG: monoacylglycerol; MGDG: monogalactosyldiacylglycerol; DGDG: digalactosyldiacylglycerol.

	L1	L2	L3	P1	P2	P3
TAG	62.7	79.6	67.5	28.6	13.3	14.3
FAIc	2.2	0.0	0.3	0.8	0.5	0.3
DAG	3.8	2.9	4.4	18.7	14.5	13.8
FFA	0.9	0.4	1.1	3.4	1.6	1.9
MAG	0.4	0.1	0.2	2.6	0.9	1.0
MGDG	26.8	15.2	23.7	36.9	62.5	64.1
DGDG	3.2	1.7	2.9	8.9	6.8	4.7

## Data Availability

Not applicable.

## References

[1] Beer L.L., Boyd E.S., Peters J.W., Posewitz M.C. (2009). Engineering algae for biohydrogen and biofuel production. Curr. Opin. Biotechnol..

[2] Yadugiri V. (2009). Milking diatoms–a new route to sustainable energy. Curr. Sci..

[3] Varfolomeev S., Wasserman L. (2011). Microalgae as source of biofuel, food, fodder, and medicines. Appl. Biochem. Microbiol..

[4] Adarme-Vega T.C., Lim D.K., Timmins M., Vernen F., Li Y., Schenk P.M. (2012). Microalgal biofactories: A promising approach towards sustainable omega-3 fatty acid production. Microb. Cell Factories.

[5] Bhattacharya M., Goswami S. (2020). Microalgae—A green multi-product biorefinery for future industrial prospects. Biocatal. Agric. Biotechnol..

[6] Beigbeder J.-B., Sanglier M., de Medeiros Dantas J.M., Lavoie J.-M. (2021). CO_2_ capture and inorganic carbon assimilation of gaseous fermentation effluents using Parachlorella kessleri microalgae. J. CO2 Util..

[7] Schiano di Visconte G., Spicer A., Chuck C.J., Allen M.J. (2019). The microalgae biorefinery: A perspective on the current status and future opportunities using genetic modification. Appl. Sci..

[8] Bibi F., Jamal A., Huang Z., Urynowicz M., Ali M.I. (2022). Advancement and role of abiotic stresses in microalgae biorefinery with a focus on lipid production. Fuel.

[9] Borowitzka M.A., Vonshak A. (2017). Scaling up microalgal cultures to commercial scale. Eur. J. Phycol..

[10] Pulz O., Gross W. (2004). Valuable products from biotechnology of microalgae. Appl. Microbiol. Biotechnol..

[11] Borowitzka M.A. (2013). High-value products from microalgae—Their development and commercialisation. J. Appl. Phycol..

[12] Chew K.W., Yap J.Y., Show P.L., Suan N.H., Juan J.C., Ling T.C., Lee D.-J., Chang J.-S. (2017). Microalgae biorefinery: High value products perspectives. Bioresour. Technol..

[13] Sirakov M., Palmieri M., Iovinella M., Davis S.J., Petriccione M., di Cicco M.R., De Stefano M., Ciniglia C. (2021). Cyanidiophyceae (Rhodophyta) Tolerance to Precious Metals: Metabolic Response to Palladium and Gold. Plants.

[14] Molazadeh M., Ahmadzadeh H., Pourianfar H.R., Lyon S., Rampelotto P.H. (2019). The use of microalgae for coupling wastewater treatment with CO_2_ biofixation. Front. Bioeng. Biotechnol..

[15] Guduru R.K., Gupta A.A., Dixit U., Khalid M., Dharaskar S.A., Sillanpää M., Siddiqui H. (2022). Biological processes for CO_2_ capture. Emerging Carbon Capture Technologies.

[16] Eilertsen H.C., Eriksen G.K., Bergum J.-S., Strømholt J., Elvevoll E., Eilertsen K.-E., Heimstad E.S., Giæver I.H., Israelsen L., Svenning J.B. (2022). Mass cultivation of microalgae: I. Experiences with vertical column airlift photobioreactors, diatoms and CO_2_ sequestration. Appl. Sci..

[17] Vonshak A. (1990). Recent advances in microalgal biotechnology. Biotechnol. Adv..

[18] Han P., Lu Q., Fan L., Zhou W. (2019). A review on the use of microalgae for sustainable aquaculture. Appl. Sci..

[19] Beijierinck M. (1890). Kulturversuche mit Zoochloren, Lichenenggonidien und anderen niederen Algen. Physis.

[20] Kok B. (1953). Experiments on photosynthesis by Chlorella in flashing light. Algal Culture from Laboratory to Pilot Plant.

[21] Nethravathy M., Mehar J.G., Mudliar S.N., Shekh A.Y. (2019). Recent advances in microalgal bioactives for food, feed, and healthcare products: Commercial potential, market space, and sustainability. Compr. Rev. Food Sci. Food Saf..

[22] García J.L., De Vicente M., Galán B. (2017). Microalgae, old sustainable food and fashion nutraceuticals. Microb. Biotechnol..

[23] Gjøsund S.H., Skjermo J., Forbord S., Jafarzadeh S., Sletta H., Aasen I.M., Hagemann A., Chauton M.S., Aursand I.G., Evjemo J.O. (2020). Bærekraftig Fôr Til Norsk Laks.

[24] Mohsenpour S.F., Hennige S., Willoughby N., Adeloye A., Gutierrez T. (2021). Integrating micro-algae into wastewater treatment: A review. Sci. Total Environ..

[25] Fozer D., Kiss B., Lorincz L., Szekely E., Mizsey P., Nemeth A. (2019). Improvement of microalgae biomass productivity and subsequent biogas yield of hydrothermal gasification via optimization of illumination. Renew. Energy.

[26] Lim Y.A., Chong M.N., Foo S.C., Ilankoon I. (2021). Analysis of direct and indirect quantification methods of CO_2_ fixation via microalgae cultivation in photobioreactors: A critical review. Renew. Sustain. Energy Rev..

[27] Ugwu C., Aoyagi H., Uchiyama H. (2008). Photobioreactors for mass cultivation of algae. Bioresour. Technol..

[28] Blanken W., Cuaresma M., Wijffels R.H., Janssen M. (2013). Cultivation of microalgae on artificial light comes at a cost. Algal Res..

[29] Ranglová K., Bureš M., Manoel J.C., Lakatos G.E., Masojídek J. (2022). Efficient microalgae feed production for fish hatcheries using an annular column photobioreactor characterized by a short light path and central LED illumination. J. Appl. Phycol..

[30] Richmond A., Richmond A., Hu Q. (2013). Biological principles of mass cultivation of photoautotrophic microalgae. Handbook of Microalgal Culture: Applied Phycology and Biotechnology.

[31] Geider R., Osborne B. (1987). Light absorption by a marine diatom: Experimental observations and theoretical calculations of the package effect in a small Thalassiosira species. Mar. Biol..

[32] Marañón E., Steele J., Thorpe A., Turekian K. (2009). Phytoplankton size structure. Marine Biology: A Derivative of the Encyclopedia of Ocean Sciences.

[33] Sinclair D. (1947). Light scattering by spherical particles. JOSA.

[34] Baker E.T., Lavelle J.W. (1984). The effect of particle size on the light attenuation coefficient of natural suspensions. J. Geophys. Res. Ocean..

[35] Agustí S. (1991). Light environment within dense algal populations: Cell size influences on self-shading. J. Plankton Res..

[36] Nelson N.B., Prézelin B.B., Bidigare R.R. (1993). Phytoplankton light absorption and the package effect in California coastal waters. Mar. Ecol. Prog. Ser..

[37] Tennessen D.J., Bula R.J., Sharkey T.D. (1995). Efficiency of photosynthesis in continuous and pulsed light emitting diode irradiation. Photosynth. Res..

[38] Lunka A.A., Bayless D.J. (2013). Effects of flashing light-emitting diodes on algal biomass productivity. J. Appl. Phycol..

[39] Schulze P.S., Guerra R., Pereira H., Schüler L.M., Varela J.C. (2017). Flashing LEDs for microalgal production. Trends Biotechnol..

[40] Eilertsen H.C., Holm-Hansen O. (2000). Effects of high latitude UV radiation on phytoplankton and nekton modelled from field measurements by simple algorithms. PolarRes.

[41] Utermöhl H. (1958). Zur Vervollkommnung der quantitativen Phytoplanktonmethodik. Mitt. Int. Ver. Theor. Angew. Limnol..

[42] Hasle G.R., Syvertsen E.E., Tomas C.R. (1997). Marine Diatoms. Identifying Marine Phytoplankton.

[43] Menden-Deuer S., Lessard E.J. (2000). Carbon to volume relationships for dinoflagellates, diatoms, and other protist plankton. Limnol. Oceanogr..

[44] Guillard R.R.L., Ryther J.H. (1962). Studies of marine plankton diatoms. I. Cyclotella nana Hustedt and Detonula confervacea (Cleve) Gran. Can. J. Microbiol..

[45] Jensen I., Mæhre H., Tømmerås S., Eilertsen K., Olsen R., Elvevoll E. (2012). Farmed Atlantic salmon (*Salmo salar* L.) is a good source of long chain omega-3 fatty acids. Nutr. Bull..

[46] Folch J., Lees M., Stanley G.S. (1957). A simple method for the isolation and purification of total lipides from animal tissues. J. Biol. Chem..

[47] Cequier-Sánchez E., Rodriguez C., Ravelo A.G., Zarate R. (2008). Dichloromethane as a solvent for lipid extraction and assessment of lipid classes and fatty acids from samples of different natures. J. Agric. Food Chem..

[48] Abreu S., Solgadi A., Chaminade P. (2017). Optimization of normal phase chromatographic conditions for lipid analysis and comparison of associated detection techniques. J. Chromatogr..

[49] Platt T., Jassby A.D. (1976). The relationship between photosynthesis and light for natural assemblages of coastal marine phytoplankton 1. J. Phycol..

[50] Platt T., Gallegos C., Harrison W.G. (1982). Photoinhibition of photosynthesis in natural assemblages of marine phytoplankton. J. Mar. Res..

[51] Yale C., Forsythe A.B. (1976). Winsorized regression. Technometrics.

[52] Malerba M.E., Palacios M.M., Palacios Delgado Y.M., Beardall J., Marshall D.J. (2018). Cell size, photosynthesis and the package effect: An artificial selection approach. New Phytol..

[53] Marañón E. (2015). Cell size as a key determinant of phytoplankton metabolism and community structure. Annu. Rev. Mar. Sci..

[54] Strickland J.D.H., Parsons T.R., Stevenson J.C. (1972). A Practical Handbook of Seawater Analysis.

[55] Webb W.L., Newton M., Starr D. (1974). Carbon dioxide exchange of Alnus rubra: A mathematical model. Oecologia.

[56] Dubinsky Z., Falkowski P.G., Post A.F., Van Hes U.M. (1987). A system for measuring phytoplankton photosynthesis in a defined light field with an oxygen electrode. J. Plankton Res..

[57] Ryther J., Vaccaro R. (1954). A comparison of the oxygen and 14C methods of measuring marine photosynthesis. ICES J. Mar. Sci..

[58] Williams P., Raine R. (1979). Agreement between the c-14 and oxygen methods of measuring phytoplankton production-reassessment of the photosynthetic quotient. Oceanol. Acta.

[59] Trampe E., Hansen P.J., Kuhl M. (2015). A comparison of photosynthesis measurements by O_2_ evolution, 14 C assimilation, and variable chlorophyll fluorescence during light acclimatization of the diatom Coscinodiscus granii. Algae.

[60] Kim N.-J., Lee C.-G. (2001). A theoretical consideration on oxygen production rate in microalgal cultures. Biotechnol. Bioprocess Eng..

[61] Palanisamy K.M., Rahim M.H.A., Govindan N., Ramaraj R., Kuppusamy P., Maniam G.P. (2022). Effect of blue light intensity and photoperiods on the growth of diatom Thalassiosira pseudonana. Bioresour. Technol. Rep..

[62] Wagner H., Jakob T., Wilhelm C. (2006). Balancing the energy flow from captured light to biomass under fluctuating light conditions. New Phytol..

[63] del Pilar Sánchez-Saavedra M., Voltolina D. (1996). Effect of blue-green light on growth rate and chemical composition of three diatoms. J. Appl. Phycol..

[64] Schellenberger Costa B., Jungandreas A., Jakob T., Weisheit W., Mittag M., Wilhelm C. (2013). Blue light is essential for high light acclimation and photoprotection in the diatom Phaeodactylum tricornutum. J. Exp. Bot..

[65] Stauber J.L., Jeffrey S. (1988). Photosynthetic pigments in fifty—One species of marine diatoms. J. Phycol..

[66] Ogbonna J.C., Yada H., Tanaka H. (1995). Light supply coefficient: A new engineering parameter for photobioreactor design. J. Ferment. Bioeng..

[67] Perner-Nochta I., Posten C. (2007). Simulations of light intensity variation in photobioreactors. J. Biotechnol..

[68] Nymark M., Valle K.C., Brembu T., Hancke K., Winge P., Andresen K., Johnsen G., Bones A.M. (2009). An integrated analysis of molecular acclimation to high light in the marine diatom Phaeodactylum tricornutum. PLoS ONE.

[69] Gilstad M., Sakshaug E. (1990). Growth rates of ten diatom species from the Barents Sea at different irradiances and day lengths. Mar. Ecol. Prog. Ser..

[70] Croteau D., Lacour T., Schiffrine N., Morin P.I., Forget M.H., Bruyant F., Ferland J., Lafond A., Campbell D.A., Tremblay J.É. (2022). Shifts in growth light optima among diatom species support their succession during the spring bloom in the Arctic. J. Ecol..

[71] Falkowski P.G. (1980). Light-shade adaptation in marine phytoplankton. Primary Productivity in the Sea.

[72] Lavaud J. (2007). Fast regulation of photosynthesis in diatoms: Mechanisms, evolution and ecophysiology. Funct. Plant Sci. Biotechonology.

[73] Rascher U., Nedbal L. (2006). Dynamics of photosynthesis in fluctuating light. Curr. Opin. Plant Biol..

[74] Smith R.C., Wilson W.H. (1972). Photon scalar irradiance. Appl. Opt..

[75] Eppley R.W. (1972). Temperature and phytoplankton growth in the sea. Fish. Bull..

[76] Huseby S., Degerlund M., Eriksen G.K., Ingebrigtsen R.A., Eilertsen H.C., Hansen E. (2013). Chemical diversity as a function of temperature in six northern diatom species. Mar. Drugs.

[77] Iluz D., Alexandrovich I., Dubinsky Z. (2012). The enhancement of photosynthesis by fluctuating light. Artif. Photosynth..

[78] Terry K.L. (1986). Photosynthesis in modulated light: Quantitative dependence of photosynthetic enhancement on flashing rate. Biotechnol. Bioeng..

[79] Vejrazka C., Janssen M., Streefland M., Wijffels R.H. (2011). Photosynthetic efficiency of Chlamydomonas reinhardtii in flashing light. Biotechnol. Bioeng..

[80] Roselló Sastre R.M. (2010). Kopplung Physiologischer und Verfahrenstechnischer Parameter Beim Wachstum und Bei der Produktbildung der Rotalge Porphyridium Purpureum.

[81] Mouget J.-L., de la Noë J., Legendre L., Jean Y., Viarouge P. (1995). Long-term acclimatization of Scenedesmus bicellularis to high-frequency intermittent lighting (100 Hz). I. Growth, photosynthesis and photosystem II activity. J. Plankton Res..

[82] Goss R., Lepetit B. (2015). Biodiversity of NPQ. J. Plant Physiol..

[83] Hüner N.P., Bode R., Dahal K., Hollis L., Rosso D., Krol M., Ivanov A.G. (2012). Chloroplast redox imbalance governs phenotypic plasticity: The “grand design of photosynthesis” revisited. Front. Plant Sci..

[84] Allahverdiyeva Y., Suorsa M., Tikkanen M., Aro E.-M. (2015). Photoprotection of photosystems in fluctuating light intensities. J. Exp. Bot..

[85] Laufs U., Parhofer K.G., Ginsberg H.N., Hegele R.A. (2020). Clinical review on triglycerides. Eur. Heart J..

[86] Bovet P., Faeh D., Madeleine G., Viswanathan B., Paccaud F. (2007). Decrease in blood triglycerides associated with the consumption of eggs of hens fed with food supplemented with fish oil. Nutr. Metab. Cardiovasc. Dis..

[87] Olvera-Gonzalez E., Escalante-Garcia N., Myers D., Ampim P., Obeng E., Alaniz-Lumbreras D., Castaño V. (2021). Pulsed led-lighting as an alternative energy savings technique for vertical farms and plant factories. Energies.

[88] Valegård K., Andralojc P.J., Haslam R.P., Pearce F.G., Eriksen G.K., Madgwick P.J., Kristoffersen A.K., van Lun M., Klein U., Eilertsen H.C. (2018). Structural and functional analyses of Rubisco from arctic diatom species reveal unusual posttranslational modifications. J. Biol. Chem..

[89] Barry A.N., Starkenburg S.R., Sayre R.T. (2015). Strategies for optimizing algal biology for enhanced biomass production. Front. Energy Res..

